# Sustainable miniaturized smartphone-coupled TLC platform for innovative cleaning validation: application to tizanidine combinations under challenging concentration ratios

**DOI:** 10.1038/s41598-026-62219-6

**Published:** 2026-07-19

**Authors:** Mariam O. Abd el-Aziz, Ahmed H. Nadim, Hany H. Monir, M. Nebsen, Sameh E. Younis

**Affiliations:** 1 Department of Pharmaceutical Chemistry, Faculty of Pharmacy, Pharos University in Alexandria, Canal El Mahmoudia Street, Beside Green Plaza Complex, Alexandria, 21648 Egypt; 2 Pharmaceutical Analytical Chemistry Department, Faculty of Pharmacy, Cairo University, Kasr El-Aini Street, Cairo, 11562 Egypt

**Keywords:** Cleaning validation, Portable at-line analyzer, Smartphone-based TLC, Green analytical chemistry, Multidimensional sustainability, Tizanidine, Drug discovery, Health care, Medical research

## Abstract

Cross-contamination in pharmaceutical industry is a critical threat to patient safety, potentially reducing therapeutic efficacy and causing adverse effects and addiction. Drugs with narrow therapeutic index and high abuse potential are high-risk candidates, such as tizanidine (TZN). Its carryover may trigger harmful effects, withdrawal symptoms, and unintended dependence. Although cleaning validation minimizes these risks, rapid and reliable tools are still needed to support routine monitoring. To address this need, a portable at-line analytical platform was developed for TZN determination in combination with aceclofenac (ACF), ibuprofen (IBF), and paracetamol (PRC). It employs FDA-recommended swabbing approach, followed by smartphone-based TLC coupled with Color Analyzer and ImageJ for image processing. It provides a rapid, simple, and eco-friendly on-site tool for cleaning validation, enabling both quantitative analysis and qualitative screening via color density charts; a combination not achieved by reported methods. The mobile phase was optimized as toluene-acetone-ethanol (2.3:2.7:0.6 v/v/v), and smartphone-based analysis parameters were optimized. Validation according to ICH guidelines demonstrated good linearity and high sensitivity for the investigated drugs, with quantitation limits below established acceptance limits. Multidimensional sustainability was confirmed using moGAPI, CACI, VIGI, and RGB-12 assessments, highlighting the method as a novel sustainable tool for reliable cleaning validation of TZN combinations.

**Supplementary Information:**

The online version contains supplementary material available at 10.1038/s41598-026-62219-6.

## Introduction

Cross-contamination refers to the accidental transfer of residues from one product to the subsequent batches of another during the manufacturing process^1^. Its likelihood in multi-product pharmaceutical manufacturing facilities may pose serious hazards to patient safety. The carryover of residual contaminants has serious effects on treatment efficacy. In addition, it may result in adverse drug reactions or toxicity, particularly in drugs with narrow therapeutic index. In some cases, the risk of unintended dependence may increase if the residues originate from potentially abused drugs^2^. Although tizanidine (TZN) is a widely used muscle relaxant, its carry over may cause harmful effects and poses potential for abuse^3^.

Chemically, TZN is 5-chloro-N-(4,5-dihydro-1 H-imidazol-2-yl)2,1,3-benzothiadiazol-4-amine hydrochloride^4^. It is commercially available in single-ingredient formulations containing 2.0 and 4.0 mg. Its 2.0 mg dose is also formulated in fixed-dose combinations with aceclofenac (ACF) 100 mg, ibuprofen (IBF) 400 mg, or paracetamol (PRC) 500 mg, among other available combinations^5,6^. TZN is a centrally acting α-2 adrenergic receptor agonist. It acts by inhibiting the presynaptic motor neurons, together with the spinal inter-neuronal activity, thus producing anti-nociceptive and anticonvulsant activities^7^. It is widely prescribed as a fast-acting anti-spastic drug in conditions such as multiple sclerosis, spinal cord or brain injuries^8^. However, it is only recommended as a short-term treatment to avoid withdrawal symptoms upon abrupt discontinuation^3,9^. Its narrow therapeutic window may increase the likelihood of CVS and CNS adverse effects, as well as hepatotoxicity. Moreover, its sedative and hallucination effects may contribute to the risk of physical dependence and potential misuse^9^. Consequently, cross-contamination by TZN during pharmaceutical manufacturing may lead to unexplained and unexpected withdrawal symptoms, adverse reactions, and potential abuse, in patients receiving other medications known for their history of safe use.

Cleaning validation is a cornerstone to prevent the risk of carryover and its subsequent hazardous effects. FDA and ICH guidance for industry consider cleaning validation a critical component of current good manufacturing practice (cGMP)^10,11^. It has to be rigorously inspected to verify the effectiveness of the cleaning process in residues removal from the equipment to a predetermined limit. Although regulatory authorities have not established acceptance limits for contaminants, they endorse the use of maximum allowable carryover (MACO), to determine scientifically justified limits for each contaminant^12^. MACO is the maximum amount of the drug considered safe to be transferred to the subsequent formulation^13^. It is determined based on doses of previous and next products, batch size, route of administration, and the employed sampling method^12^. Effective cleaning validation can be achieved by employing a suitable sampling technique and an appropriate analytical method^14^. The sampling of equipment parts is performed using either swabbing or rinsing approaches, guided by the ‘worst-case’ scenario regarding the investigated drug properties, and the equipment design. The swab sampling is preferred for the poorly water soluble drugs as swabs can be targeted directly to high-risk surface areas. In contrast, the rinse sampling is applied when swabbing is impractical, such as for large and inaccessible equipment parts^15^. The FDA therefore recommends the swabbing approach whenever suitable, as it provides more specific residue assessment^10^.

Proper analytical method is employed to assay residual drug levels in light of the predetermined MACO. Analytical methods that fit for cleaning validation purposes should demonstrate reliability, accuracy, specificity, and robustness. Also, they have to be rapid to enable a routine monitoring without delaying the manufacturing operations, and simple enough to minimize the dependence on highly qualified personnel. In addition, they should be cost- and energy-efficient, to comply with the economic and environmental measures. Moreover, their potential integration into portable at-line devices, would facilitate the on-site and real-time auditing. Conventional analytical methods reported in the literature of cleaning validation include total organic carbon (TOC)^16^, HPLC^1,17^, UPLC^18,19^, and HPTLC^14^. Such methods offer high sensitivity and accuracy. TOC method is also rapid. However, it randomly assays the total carbon residues, irrespective of their origin, so, it lacks specificity^20^. Chromatography-based methods provide high specificity. However, they require capital-intensive instrumentation, relatively high energy consumption, trained personnel, and samples transportation from the collection sites to laboratories. Accordingly, there remains a need for an analytical strategy that retains the strengths of conventional methods while enabling a portable at-line analytical platform, inspired by the concept of point-of-care testing (POCT), for application in the cleaning validation field. It must be considered that, to date, no analytical cleaning validation method has yet been reported for TZN either in single or combined formulations.

Smartphone-based analytical methods are rapidly gaining high recognition as valuable alternative to conventional techniques. They have comparable performance while meeting the key requirements of modern analytical chemistry including portability, accessibility, cost-efficiency and environmental friendliness^21^. These methods have been successfully applied in various fields including clinical diagnosis^22^, therapeutic drug monitoring^23^, substances abuse detection^24^, authenticity characterization^25^, food analysis^26^, environmental monitoring^27^, and pharmaceutical analysis^28^. Nevertheless, smartphone-based methods have not yet been fully exploited in the field of cleaning validation. In this context, integrating smartphone technology with thin layer chromatography (TLC) technique presents a promising approach for developing a reliable portable at-line analyzer. TLC; a fingerprint based analytical method, facilitates the analysis of multi-component formulations, together with the rapid acquisition of qualitative information for each substance^29^. Smartphone-based TLC technique utilizes the smartphone’s camera to capture images of the developed TLC plates, after their visualization by using UV-lamps or staining reagents. These images are simultaneously processed using various color models through dedicated software that translates the pixel values into numerical color intensities^25,30^. At this stage, a color-key chart supported with calibration data can be exerted. On this basis, a portable at-line analyzer can be developed for qualitative and quantitative cleaning validation. However, prior to implementation, the proposed approach has to demonstrate compliance with environmental metrics.

Greenness and sustainability have become pivotal considerations in modern analytical chemistry. Green analytical chemistry (GAC) is concerned with promoting safer practices for human and environment regarding the utilized reagents and instruments^31^. White analytical chemistry (WAC) expands beyond these principles to consider broader sustainability measures including analytical performance and practicality^32,33^. These practices involve replacing hazardous solvents with safer alternatives, minimizing reagents and samples consumption, considering proper waste treatment or recycling strategies. In addition, depending on portable and miniaturized instruments to further conserve resources as they require less chemicals amounts, and lower energy input^34^. Several environmental metrics have been developed to assess analytical method’s greenness; as modified green analytical procedure index (moGAPI)^35,36^, and whiteness; as RGB-12 algorithm^34,37^. Additional metrics address specific aspects of sustainability include click analytical chemistry index (CACI) that primarily targets the method’s practicality^38^, and violet innovation grade index (VIGI) for the innovation degree^39^. Hence, combining the conventional validation procedures with the appropriate environmental assessment metrics, is essential to provide a comprehensive evaluation of any proposed analytical approach.

The current study aims to introduce a portable at-line analytical platform to the cleaning validation field for the first time. A smartphone-based TLC approach is proposed as a practical candidate to assemble the portable at-line analyzer, that is designed to enable a rapid on-site screening of potential contaminants using the attached color density charts, along with a reliable quantitative analysis. Two image-processing strategies are incorporated and comparatively evaluated. They involve a mobile application (Color Analyzer) and a widely used software (ImageJ). Moreover, this analyzer is applied for the first time for the determination of swab samples containing TZN in combination with ACF, IBF, and PRC. The method is optimized and validated for each drug, to ensure its applicability on the commercially available TZN combinations exhibiting challenging dose ratios. Finally, the multidimensional sustainability profile of the proposed analyzer is assessed using moGAPI, CACI, VIGI, and RGB-12 metrics.

## Materials and methods

### Instruments and software

TLC separation was carried out on pre-coated silica gel F_254_ TLC plates (50.0 mm x 50.0 mm, 0.25 mm, Merck, Darmstadt, Germany). Plate development occurred in 500-mL glass beaker sealed with elastic parafilm, to provide a clear and sealed environment. The developed plates were visualized at a wavelength of 254 nm emitted by UV-lamp (Spectroline, New York, USA). Images were captured using the built-in rear camera of Xiaomi Redmi Note 13 smartphone. It features a triple camera system consisting of a 108 MP main camera (f/1.755), an 8 MP ultra-wide camera (f/2.2), and a 2 MP macro camera (f/2.4). Both UV visualization and image capturing were conducted inside a customized portable imaging wooden box (15.0 cm x 15.0 cm x 15.0 cm), to provide a controlled environment. It was designed with two openings (7.5 cm x 2.0 cm) at the top and front sides to accommodate the smartphone and the UV-lamp, respectively. Two imaging boxes with white and black backgrounds were constructed to optimize imaging conditions. Image processing was accomplished using the ‘Color Analyzer’, an android application that was installed from Google Play Store, and ‘ImageJ’, a computer-based software developed by the National Institutes of Health (NIH). The assessment of the multidimensional sustainability profile of the proposed approach was accomplished through the available software of moGAPI (bit.ly/MoGAPI), CACI (bit.ly/CACI2025), VIGI (bit.ly/VIGItool), and previously published Excel spreadsheet template for RGB-12^40^.

### Chemicals and reagents

Tizanidine (TZN, 99.26%) and aceclofenac (ACF, 99.00%) were obtained as a gift from Amoun Pharmaceutical Co., Qalyubia, Egypt. Ibuprofen (IBF, 98.50%) and paracetamol (PRC, 98.00%) were supplied by Pharco Pharmaceuticals, Alexandria, Egypt. The purity percentages of all investigated drugs were those specified in the manufacturers’ certificates of analysis. Analytical-grade ethanol, toluene, acetone, hexane, ethyl acetate, butanol, acetonitrile, methanol, and acetic acid were purchased from El-Nasr Chemical Co., Alexandria, Egypt. Freshly prepared distilled water was used throughout the experiments.

### Preparation of stock standard solutions and synthetic mixtures

Stock standard solutions of TZN (1.0 mg/mL), ACF (70 mg/mL), IBF (100 mg/mL), and PRC (120 mg/mL) were prepared in ethanol and stored under refrigerated conditions. Synthetic mixtures were prepared by serial dilution using the same solvent to obtain concentration ratios corresponding to the labelled strengths of various commercially available combination products. The labelled strengths of TZN (2 mg), ACF (100 mg), IBF (400 mg), and PRC (500 mg) yield a dose ratio of 1:50:200:250, respectively.

### Construction of calibration curve in swab samples

#### Preparation of swab samples

One milliliter of the synthetic mixtures containing serial concentration ranges (mg/mL) of TZN (0.02–0.17), ACF (1.0–8.5), IBF (4.0–34.0), and PRC (5.0–42.5) were prepared. The mixtures were spread onto a series of stainless-steel bowls (surface area: 100 cm^2^ each) and evaporated to dryness, leaving the corresponding residues. Cotton swabs of consistent size (2.0 cm x 2.0 cm, 90 mg) were prepared for residue collection. Prior to swabbing, each swab was moistened with the extraction solvent (500 µL ethanol). For drug extraction, the swab was held between the thumb and the index finger to apply consistent pressure to the surface during swabbing. Each bowl surface was then wiped three times using a single swab in one direction from the outer edge to the center as recommended by the standardized motion protocol^41^. The swab was then placed in a syringe and repeatedly squeezed three times using the plunger to expel the extract into a clean container, to reach maximum recovery^42^. The final volume of the obtained extract was adjusted to 1-mL with ethanol prior to analysis.

#### Chromatographic conditions

A mixture of toluene, acetone, and ethanol (2.3: 2.7: 0.6, v/v/v) was used as the mobile phase. The developing chamber was saturated with 5.6 mL of the mobile phase for 10 min at room temperature, without the use of filter paper, before plate development. Silica gel F_254_ TLC plates (50 mm x 50 mm) were used as stationary phase. Spotting was performed using a micropipette to apply a single spot of 10 µL with a width of 4.0 mm for each mixture, at 5.0 mm from the lower edge of the plate, maintaining a distance of 7.0 mm between spots. The spots were allowed to dry completely before development. The plate was then placed in the pre-saturated chamber and developed in ascending mode until the solvent front migrated 40.0 mm from the spotting line. The development was carried out for 5.0 min at ambient temperature and humidity. Finally, the developed plate was air-dried.

#### Image acquisition and processing

**Fig. 1:**
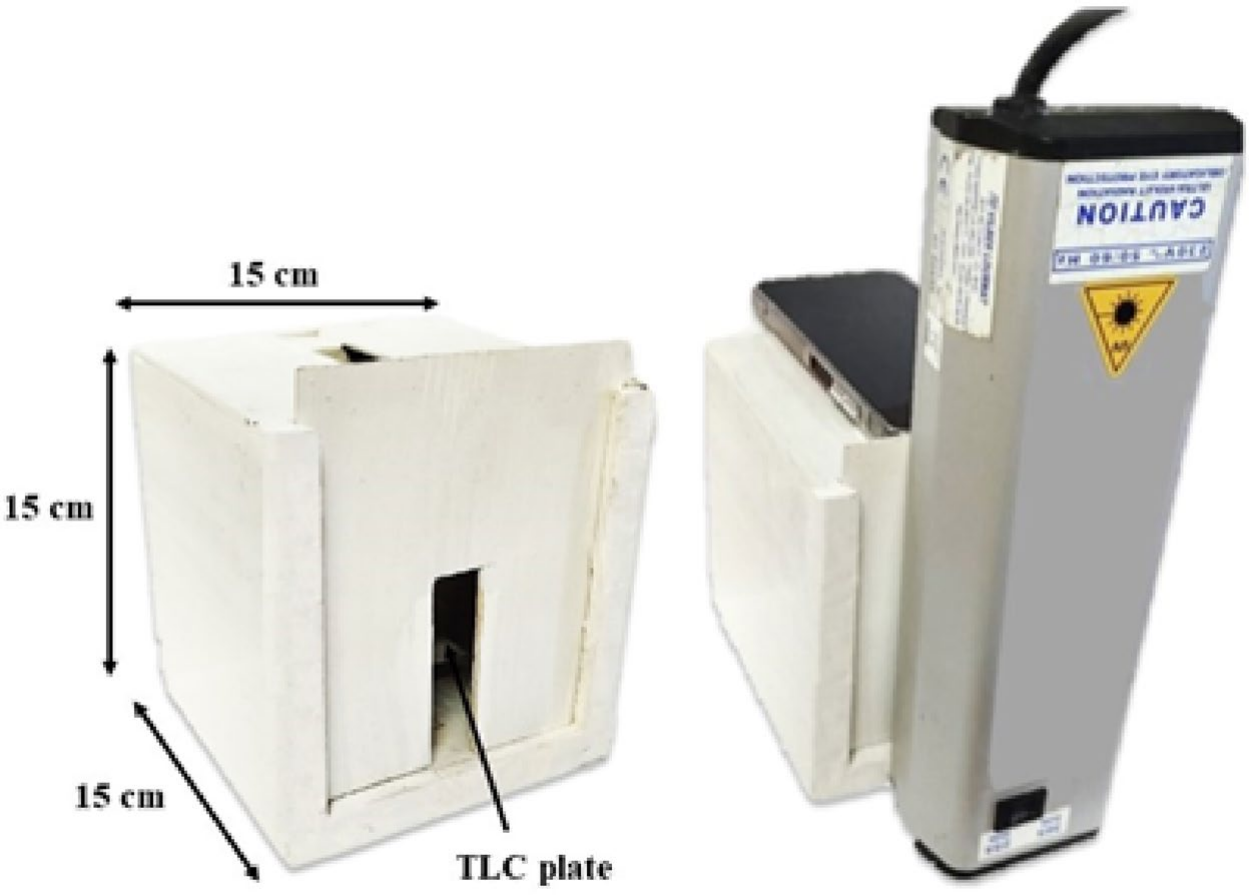
Portable wooden imaging box with a UV-lamp (254 nm) and the TLC plate horizontally placed at the center at a distance of 11.0 cm from the smartphone camera.

The developed TLC plate was horizontally placed at the center of the portable imaging box with the white background. The smartphone camera was positioned at the top opening of the box, maintaining a fixed distance of 11.0 cm from the TLC plate. The plate was visualized using a UV-lamp (254 nm) fitted at the front opening of the box (Fig. 1). Under the standardized image acquisition conditions, all images were captured at a resolution of 3000 × 4242 pixels using the smartphone’s main camera (5.0 mm focal length). The camera settings were an exposure time of 1/10 s, ISO 640, and automatic white balance. Images were acquired using the camera’s standard photo mode with all image enhancement features disabled.

Two image processing methods were applied. In the first method, the captured images were opened through Color Analyzer application, and the cursor was pointed at the center of each spot. The green channel intensity of the RGB color model was recorded for each spot, reflecting the corresponding concentration of each mixture component. Accordingly, color density charts were generated for each drug. Whereas in the second method, images were processed using ImageJ software, and the entire spot’s area was framed using the oval selection tool. The green intensities were then obtained by applying the ‘RGB Measure’ command in the ‘Plugins’ tab. In addition, the corresponding chromatogram was attained to demonstrate the retention factor (R_f_) of the mixture components, along with the resolution of the adjacent peaks.

The individual calibration curves were constructed for each drug. The green channel intensities obtained from both methods were plotted against the corresponding concentrations (mg/mL) in the swab samples.

## Results and discussion

A portable at-line analytical platform for cleaning validation applications was developed based on a smartphone-based TLC method. Critical factors impacting the effectiveness of the cleaning validation method were evaluated including the sampling technique and the acceptance limits establishment. In addition, TLC separation together with smartphone-based analysis conditions were optimized. The method was subsequently validated, and its multidimensional sustainability profile was assessed.

### Sampling technique and calculations of maximum allowable carryover (MACO)

The assayed drugs demonstrate poor water solubility (ACF, IBF, and PRC), and sometimes a pH dependent water solubility (TZN)^43–46^. Thus, swabbing is preferred over the rinsing technique; as recommended by FDA^10^. Therefore, it was adopted to enable direct targeting of surfaces piled up with these drugs residues that cannot be rinsed with water.

MACO establishes the acceptance limits (AL) on which the cleaning validation method is based. None of the assayed drugs has an official FDA acceptance limit. So that, MACO along with the corresponding AL were estimated for each drug using the reported equations (Eqs. 1 and 2)^12^, and were presented in supplementary table S1. The input variables adopted in the calculations were selected based on the manufacturing circumstances of the investigated drugs combinations (Supplementary Table S1). The established AL (mg/mL) were 1.52, 76.22, 304.88, and 381.10 for TZN, ACF, IBF, and PRC; respectively.

1 $$\:\mathrm{M}\mathrm{A}\mathrm{C}\mathrm{O}\:\left(\mathrm{m}\mathrm{g}\right)=\frac{TD}{SF} \times \frac{BS}{LDD}$$

2 $$\:\mathrm{A}\mathrm{L}\:(\mathrm{m}\mathrm{g}/\mathrm{m}\mathrm{L})\:=\:\frac{MACO\: \times\:R\: \times\:As\: \times\:F}{At\: \times\:V}$$

Where, *TD* is the minimal therapeutic dose of the investigated drug (mg), *SF* is the safety factor, *BS* is the smallest batch size of the next drug (mg), *LDD* is the largest daily dose of the next drug (mg), *R* is the theoretical recovery factor, *As* and *At* are the swab and total surface areas (cm^2^), respectively, *F* is the dilution factor, and V is the extraction volume (mL).

### Optimization of the mobile phase for TLC separation

**Fig. 2:**
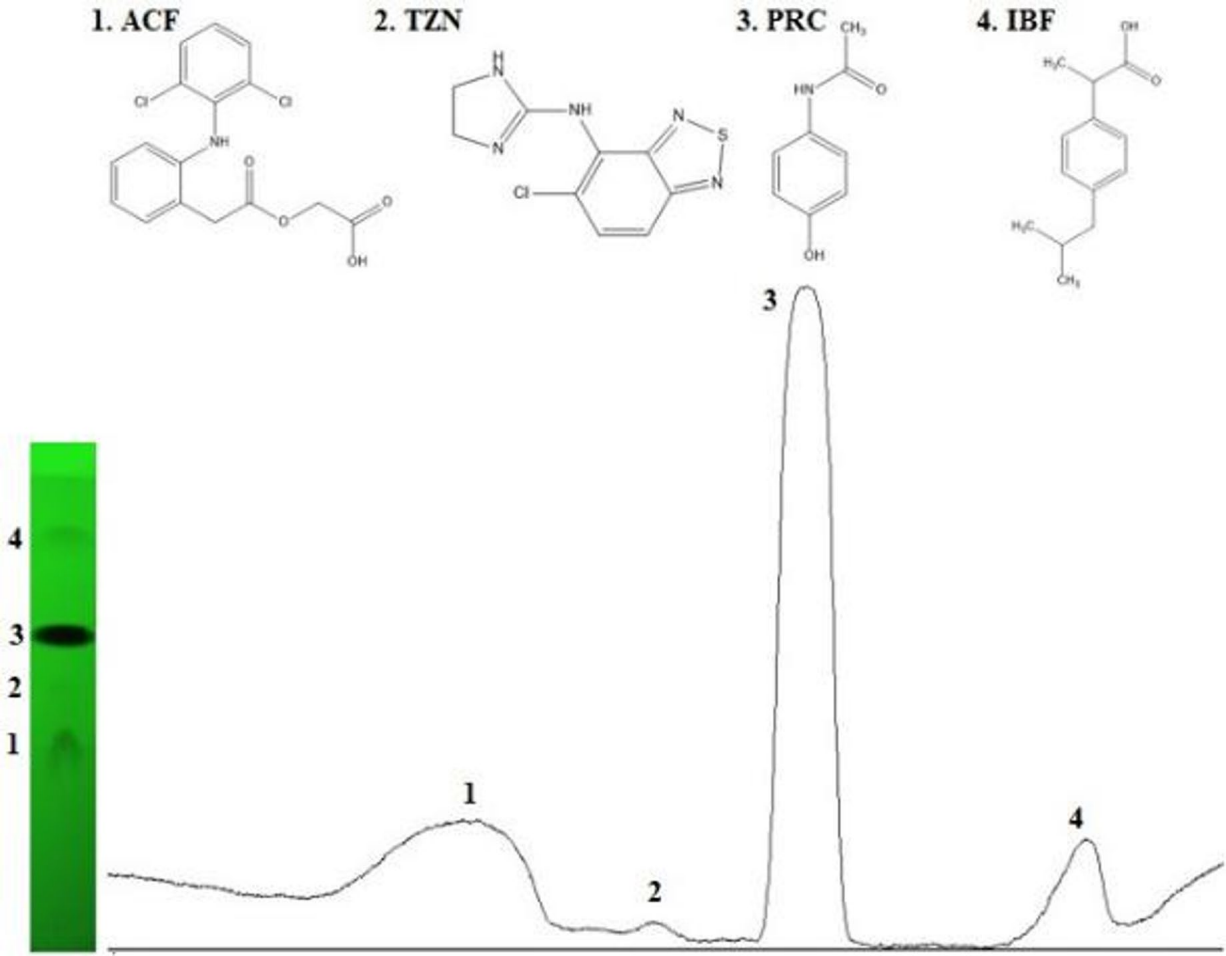
ImageJ generated TLC chromatogram of ACF (4.0 mg/mL), TZN (0.08 mg/mL), PRC (20.0 mg/mL), and IBF (16.0 mg/mL) in their swab sample applied as a 10 µL single spot.

Optimization of the mobile phase composition is critical for achieving efficient separation and selective determination of analytes in complex mixtures. Several chromatographic systems of varying polarity were investigated using different combinations of hexane, toluene, ethyl acetate, acetone, butanol, acetonitrile, ethanol, methanol, acetic acid, and water. Multiple trials demonstrated that a sharp increase or decrease in polarity resulted in inadequate separation. This was reflected in rapid migration of the PRC spot toward the solvent front, overlap between PRC and TZN spots, and failure to achieve separation of IBF. Among the tested systems, a moderately polar mobile phase system consisted of toluene-acetone-ethanol (2.3: 2.7: 0.6 v/v/v) provided complete separation of the four assayed drugs, yielding well-resolved peaks (Fig. 2). The corresponding R_f_ values were found to be 0.39, 0.54, 0.65, and 0.87 for ACF, TZN, PRC and IBF, in order. Additionally, the resolution values between adjacent peaks ranged from 1.90 to 5.44. Consequently, this mobile phase system offered adequate separation of the four analytes and was therefore adopted for further analysis. Moreover, to ensure compliance of the utilized mobile phase with environmental and sustainability considerations, a small volume was adopted (5.6 mL) and the same mobile phase was reused for multiple runs.

### Optimization of smartphone-based analysis

Optimization of image acquisition and processing parameters is essential to provide high-quality images capable of detecting minute color variations corresponding to concentration differences of the target analytes. Image acquisition was performed using the constructed imaging box to provide controlled conditions without interference from the surrounding environment. A wood-based structure was selected, owing to its durability, sustainability, insulation properties, low environmental impact, and portability^47^. This portable imaging box was designed with two openings. The front opening was integrated with a UV-lamp (254 nm) to allow consistent illumination of the TLC plates, maintaining constant light intensity throughout all measurements. While the top opening housed the smartphone camera to enable stable capturing of images at a fixed distance from the plate. Imaging was performed using the standard photo mode with all image enhancement features disabled, so that the real color intensities can be accurately retrieved. These acquisition conditions were carefully maintained throughout the study to ensure consistency in smartphone-based analysis. Furthermore, different smartphones were tested to evaluate the reproducibility. Evaluation was performed using iPhone 7 camera (7 MP) and Huawei Y 9 camera (13 MP). The obtained results from the tested smartphones were comparable to the utilized Xiaomi Redmi Note 13 (108 MP), indicating that variations in camera specifications had negligible impact on the assay.

Various parameters were carefully studied including the color model applied in image processing, the background used during imaging, and the distance from camera lens to the plate being photographed^24^.

#### Color model

The color model provides a robust descriptor of sample images and is directly linked to the method’s reliability. RGB and grey scales are among the commonly used color models for smartphone-based analysis^48^. The optimum color model for the proposed method was selected by evaluating the sensitivity (S) and correlation (r) of each color channel in relation to the concentrations of the assayed drugs in their swab samples (Table 1a). The green channel intensities demonstrated the strongest linear correlation (higher r) for TZN, ACF, and PRC, while the grey scale showed a slightly higher r for IBF. However, the green channel provided higher sensitivity (S) for all four drugs compared to the red and blue channels as well as the grey scale. Accordingly, the green channel of the RGB model was selected for further analysis of TZN, ACF, IBF, and PRC in the swab samples.

#### Imaging background

The background color is crucial for obtaining reliable and high-quality images. The optimal background for image acquisition was determined by comparing images captured against white and black surfaces. The green channel intensities corresponding to the concentrations of TZN, ACF, IBF, and PRC in the swab samples were used to construct linear models. Sensitivity (S) and correlation (r) values for both backgrounds were evaluated (Table 1a). The white background was selected as it exhibited higher sensitivity and improved correlation (r).

#### Distance from smartphone camera to the plate

The distance between the smartphone camera and the plate can directly affect measurement repeatability and overall method accuracy. The ideal distance was established based on %RSD values obtained from images of a mixture of TZN, ACF, IBF, and PRC. The signals from images captured in triplicate at varying distances (7.0–13.0 cm) against white background were recorded. As shown in Table 1b, the distance of 11.0 cm showed the lowest %RSD values. This indicated enhanced repeatability for all drugs, hence, this distance was maintained during image acquisition.

**Table 1:** Optimization of parameters affecting smartphone-based TLC analysis.

a^a^										
		TZN		ACF			IBF		PRC	
		S	r	S		r	S	r	S	r
RGB scale channel	Red	0.024	0.7068	0.345		0.7574	0.076	0.9054	0.053	0.8650
	Green	0.310	0.9984	8.919		0.9950	2.156	0.9948	1.417	0.9978
	Blue	0.082	0.9516	1.152		0.7723	0.095	0.9166	0.262	0.6043
Grey scale		0.310	0.9948	5.892		0.9930	0.630	0.9981	1.165	0.9942
Imaging background	White	0.310	0.9984	8.919		0.9950	2.156	0.9948	1.417	0.9978
	Black	0.299	0.9385	3.212		0.9686	0.620	0.9818	0.316	0.9108

b^b^										
		TZN	ACF	IBF		PRC				
		%RSD								
Distance of smartphone camera to plate (cm)	7.0	4.46	4.85	1.12		10.06				
	9.0	3.64	3.97	1.94		16.92				
	11.0	0.94	0.81	1.39		4.15				
	13.0	1.02	1.54	2.06		13.77				

^a^Sensitivity (Slope) and r values were calculated within the following concentration ranges: TZN (0.02–0.17), ACF (1.0–8.5), IBF (4.0–34.0), and PRC (5.0–42.5) mg/mL.

^b^%RSD values were calculated from mean of three determinations (*n* = 3).

### Validation

The reliability and applicability of the proposed smartphone-based TLC method for the determination of TZN, ACF, IBF, and PRC in swab samples were validated in accordance with the guidelines of ICH^49^, and the active pharmaceutical ingredients committee (APIC)^50^. The analytical performance of the two image processing methods employed (Color Analyzer and ImageJ) was comparatively assessed by evaluating the required validation parameters for each assayed drug.

#### Linearity and lower range limits

**Fig. 3:**
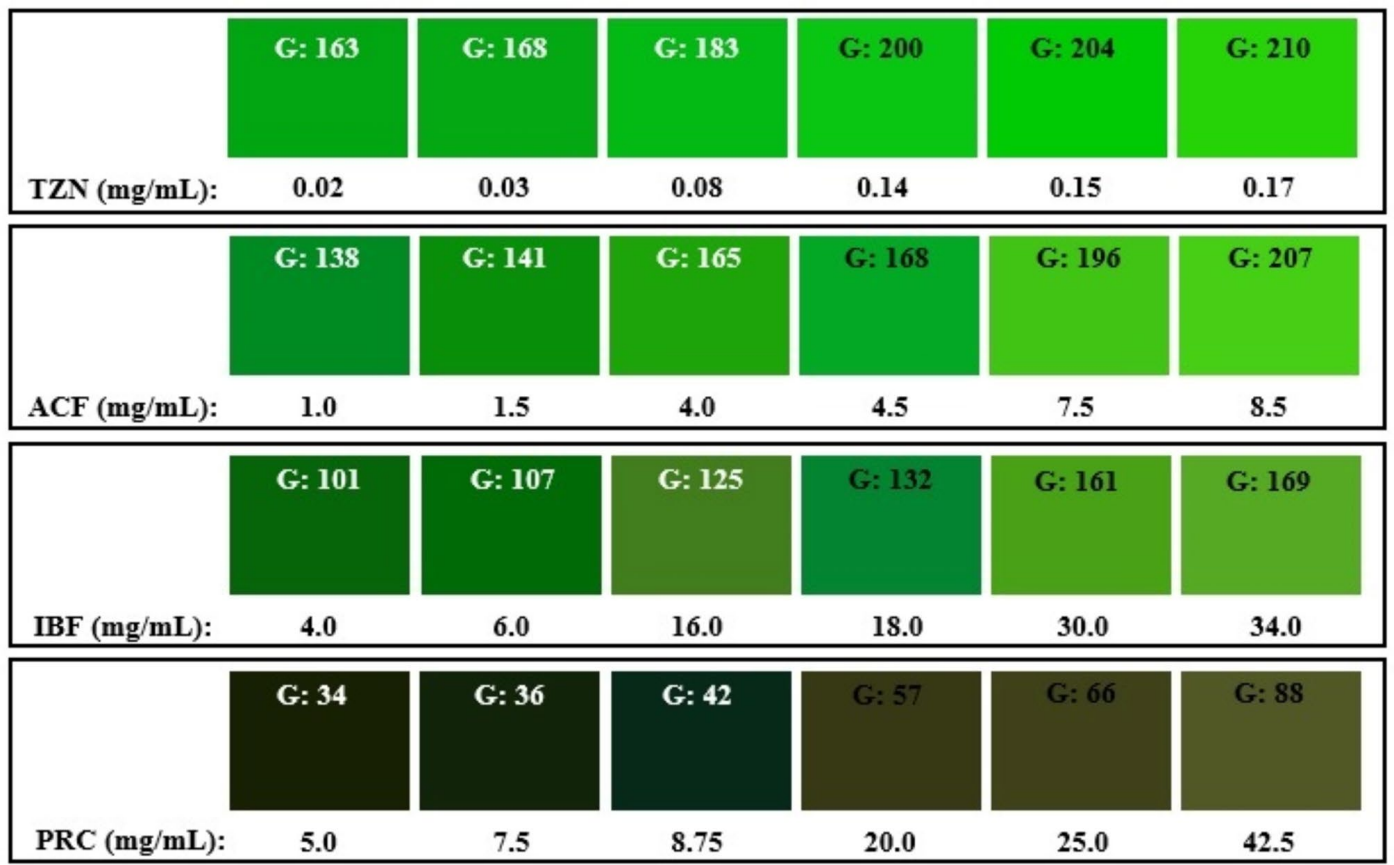
Color density charts representing different concentrations (mg/mL) of TZN, ACF, IBF, and PRC in swab samples, each applied as single 10 µL spot.

The employed concentration ranges were selected based on the pre-established AL obtained from MACO calculations for the target drugs. Six concentrations within the ranges of TZN (0.02–0.17 mg/mL), ACF (1.0–8.5 mg/mL), IBF (4.0–34.0 mg/mL), and PRC (5.0–42.5 mg/mL) were assessed using the proposed method. The green channel intensities corresponding to each drug concentrations (mg/mL in swab samples) were used to construct the calibration curves (Supplementary Figs. S1-a & S1-b). Regression analysis was performed using the least squares method. Linearity parameters including correlation coefficient, slope (b), intercept (a), standard deviation of slope (S_b_), standard deviation of intercept (S_a_), F experimental, and F critical were calculated (Table 2). All parameters successfully met the acceptance criteria. Although the ImageJ-based processing approach exhibited slightly enhanced performance for ACF, IBF, and PRC, both methods demonstrated high correlation coefficient (r ˃ 0.99), low standard deviation of slope (%S_b_), and high experimental F values relative to the critical F values for all analytes. These results indicated excellent linearity along with acceptable slope precision, representing a robust regression model with minimal residual dispersion. Furthermore, color density charts (Fig. 3) were generated from the green color intensities of the Color Analyzer-processed images to enable rapid and at-line quantitative screening of the target drugs.

Detection limits (DL) and quantitation limits (QL) were calculated (Eqs. 3 and 4)^49^, and represented in Table 2. All QL values were noticeably lower than the corresponding pre-established AL values of 1.52, 76.22, 304.88, and 381.10 mg/mL for TZN, ACF, IBF, and PRC, respectively. This level of sensitivity ensures the capability of the proposed smartphone-based TLC method to be reliably applied for the cleaning validation of the investigated drugs.

3 $$\:\mathrm{D}\mathrm{L}\:(\mathrm{m}\mathrm{g}/\mathrm{m}\mathrm{L})\:=\:\frac{3.3\: \times\:\sigma\:}{S}$$

4 $$\:\mathrm{Q}\mathrm{L}\:(\mathrm{m}\mathrm{g}/\mathrm{m}\mathrm{L})\:=\:\frac{10\: \times\:\sigma\:}{S}$$

Where, *σ* is the standard deviation of the response, and *S* is the slope of the calibration curve.

**Table 2:** Regression parameters of the proposed smartphone-based TLC combined with Color Analyzer and ImageJ methods for TZN, ACF, IBF, and PRC determination in swab samples.

Parameter	Smartphone-based TLC-Color Analyzer				Smartphone-based TLC-ImageJ			
	TZN	ACF	IBF	PRC	TZN	ACF	IBF	PRC
Linearity range (mg/mL)	0.02–0.17	1.0-8.5	4.0–34.0	5.0-42.5	0.02–0.17	1.0-8.5	4.0–34.0	5.0-42.5
DL (mg/mL)	0.007	0.30	2.09	3.17	0.008	0.26	1.41	1.59
QL (mg/mL)	0.02	0.90	6.34	9.60	0.02	0.79	4.28	4.83
Correlation coefficient (r)	0.9993	0.9994	0.9982	0.9963	0.9990	0.9995	0.9992	0.9991
Slope ± S_b_	0.30 ± 0.01	9.19 ± 0.16	2.27 ± 0.07	1.46 ± 0.06	0.30 ± 0.01	9.31 ± 0.14	2.26 ± 0.04	1.46 ± 0.03
Intercept ± S_a_	157.95 ± 0.67	127.79 ± 0.83	91.66 ± 1.44	27.43 ± 1.40	158.75 ± 0.76	125.43 ± 0.74	93.33 ± 0.97	29.09 ± 0.70
S_b_%	1.92	1.71	1.57	4.30	2.23	1.49	2.02	2.16
S _y/x_^a^	0.69	0.87	1.51	1.63	0.78	0.77	1.02	0.82
F experimental	2700	3424	1115	540	2001	4474	2446	2137
F critical	8 × 10^− 7^	5 × 10^− 7^	5 × 10^− 6^	2 × 10^− 5^	1 × 10^− 6^	3 × 10^− 7^	1 × 10^− 6^	1 × 10^− 6^

^a^S_y/x_ represents standard deviation of residuals.

#### Precision and accuracy

Intra-day and inter-day precision and accuracy of the proposed method were evaluated. The developed smartphone-based TLC method was assessed at three concentration levels across the linear range of each drug. Triplicate measurements were performed for TZN (0.02, 0.08, and 0.17 mg/mL), ACF (1.0, 4.50, and 8.50 mg/mL), IBF (4.0, 18.0, and 34.0 mg/mL), and PRC (5.0, 20.0, and 42.50 mg/mL). The %RSD and %recovery were calculated for the Color Analyzer- and ImageJ-based processing methods (Supplementary Table S2). The obtained values fell within the APIC acceptance criteria for precision (%RSD ≤ 10%) and accuracy (%Recovery = 90–110%)^50^.

#### Robustness

Robustness of the proposed method was examined under varying conditions. Evaluations included swab size (90 ± 20 mg), extraction solvent volume (500 ± 20 µL), mobile phase ratio (Toluene 2.3 ± 0.1: Acetone 2.7 ± 0.1: Ethanol 0.6 ± 0.1 v/v/v), and imaging distance (11 ± 0.3 cm). Triplicate analyses were conducted under these conditions for swabs containing mixtures of TZN (0.1 mg/mL), ACF (5.0 mg/mL), IBF (20.0 mg/mL), and PRC (25.0 mg/mL). %RSD values were calculated. All results met the acceptance criterion of %RSD ˂ 10%^50^, with maximum values of 5.38% and 5.13% for Color Analyzer and ImageJ, respectively (Supplementary Table S3).

#### Specificity

The aforementioned procedure was applied under the optimized conditions to analyze swabs from a clean stainless steel bowl, as well as swabs containing the extraction solvent, mobile phase, and common tablet excipients such as starch, talc, lactose, silicon dioxide, stearic acid, and magnesium stearate. No spots were observed for any of the tested swabs on the developed plate (Supplementary Fig. S2). This confirmed the specificity of the developed smartphone-based TLC method for the determination of TZN, ACF, IBF, and PRC, even in the presence of potential interfering substances.

#### Recovery

APIC declares that %recovery ≥ 90% is considered acceptable^50^. Swab samples containing three concentration levels of the investigated drugs were analyzed in triplicate. Their signals were compared with those obtained from standard solutions at the same concentrations. %Recoveries were calculated (Supplementary Table S4). The calculated mean %recovery values using Color Analyzer were 93.97%, 93.90%, 94.98%, and 90.37%, for TZN, ACF, IBF, and PRC, respectively. Those obtained using ImageJ were 94.41%, 94.58%, 95.53%, and 91.76%, respectively. Thus, the proposed procedure provided efficient recovery of residues from previously cleaned surfaces.

### Multidimensional sustainability profile assessment

The developed portable at-line analyzer was designed in accordance with the principles of green and white analytical chemistry, incorporating several eco-friendly practices. Quantitative multidimensional sustainability assessment metrics demonstrated improved scores compared to the previously reported methods for TZN determination in its combinations (Table 3).

#### MoGAPI metric

To assess method greenness, the moGAPI was employed. This tool evaluates methodological aspects including sampling, method type, solvents and reagents, instrumentation, and quantification capability. It combines the visual assessment of the original GAPI tool with quantitative scoring, generating red, yellow and green pictograms, and assigning a score to each criterion. A surrounding color scale and an overall percentage score indicate the method’s greenness. Methods scoring ≥ 75% with green-colored scales are considered excellent. Those scoring 50–74% with yellow-colored scales are regarded acceptable. Methods are considered inadequately green when they score ˂ 50% with red-colored scales^35^.

As demonstrated in Table 3, the proposed method achieved a moGAPI score of 76% compared to reported methods with scores ranging from 60 to 68%. This superiority can be attributed to several practices, including at-line sample collection, the use of greener solvents and reagents with reduced consumption to ˂ 10 mL and minimized waste generation, the dependence on energy-efficient smartphone with power consumption levels below 0.1 KWh. In addition, the reuse of the same mobile phase for consecutive analyses helped lessen the potential hazards associated with toluene and acetone. Consequently, according to the moGAPI metric, the proposed portable at-line analyzer is classified as an excellent green analytical platform.

#### CACI metric

To determine the practicality and applicability of the proposed method as a cleaning validation tool, the CACI was applied. This tool depends on the perspectives of sample size and preparation, feasibility, type and scope of application, portability, automation, sensitivity, and analysis time. Each perspective is assigned a score which is represented by a black, grey, or chromatic pictogram revealing poor, intermediate, or excellent practicality; respectively. In addition, a total score is calculated to provide a quantitative measure of practicality enabling rapid comparison of the proposed method with other ones. CACI scores ≥ 75% reflect high practicality. Scores between 50% and 74% indicate acceptable practicality, while scores ≤ 50% indicate insufficient practicality^38^.

As per Table 3, the proposed analytical platform achieved a CACI score of 80%. On the other hand, the reported methods did not exceed 74%. The proposed method’s applicability is supported by high sensitivity (QL ˂ 1% of the target concentration). Practicality is further demonstrated by the use of minimal sample size, readily available low-cost reagents and materials, and broad applicability across different matrices, along with the capability for concurrent analysis of multiple analytes. Notably, portability and short analysis time contribute to its superior performance. According to the CACI score, the proposed portable at-line analyzer represents a highly practical tool for cleaning validation.

#### VIGI metric

The VIGI tool was used to provide a quantitative assessment of the degree of innovation. This survey-based tool evaluates analytical systems according to the incorporation of advanced materials, sample preparation and analytical techniques, data processing tools, consideration of other white analytical chemistry metrics and regulatory recommendations, integration of miniaturization and automation, enhancement of sensitivity, and the introduction of interdisciplinary and novel research approach. The VIGI result appears in a star-shaped decagon, where each criterion is assigned a score (0, 5, or 10), yielding a total score out of 100, combined with a color saturation degree (ranging from white to deep violet), indicating increasing levels of innovation. A method with a VIGI score ≥ 50 is considered innovative^39^.

The developed analyzer introduces a fully smartphone-dependent analytical approach for cleaning validation and applied, for the first time, for the potentially abused drug TZN. The novelty of the proposed approach is supported by the obtained VIGI score of 65, compared to reported methods’ scores ranging from 25 to 45, as demonstrated in Table 3. The factors contributed to this innovation include the utilization of smartphone based-data processing tools, evaluation of multiple environmental metrics, compliance with FDA, ICH, and APIC guidelines, and the development of a miniaturized device as a novel approach for cleaning validation, with potential applications in pharmaceutical and environmental sectors. Therefore, the proposed portable at-line analyzer can be considered an innovative to highly innovative approach.

#### RGB-12 algorithm

The proposed method’s whiteness was assessed using the RGB-12 tool. It represents a normalized summary assessment of three categories (Red, Green, and Blue). It covers the 12 WAC criteria (R 1–4, G 1–4, and B 1–4) with individual category scores that may exceed 100. The final overall white score is normalized to a maximum value of 100. This tool provides both numerical and visual interpretation of the results, along with a final whiteness ranking among the reported methods^51^. Higher white scores reaching 100 are visually expressed as increased color saturation and indicate enhanced WAC compliance^34^.

The red category evaluates the method’s analytical performance involving number of analytes (R1), DL and QL (R2), precision (R3), and accuracy (R4)^34^. As represented in Table 3, the proposed method recorded a red score of 69.6, among the range of the reported methods (59.8–103.1). While the proposed method allowed simultaneous assay of four analytes in a single run (high R1), it did not achieve the highest scores in R 2–4. This may be attributed to its application in the cleaning validation field, where different regulatory guidelines apply. Noticeably, higher DL and QL values (Low R2) resulted from the high analyte concentrations used in the linear range established in accordance with MACO and AL of the assayed drugs. Although the method could not compete in R3 and R4, its precision and accuracy fully complied with the corresponding guidelines, as discussed earlier.

The green category evaluates the method’s environmental performance, in terms of reagents toxicity (G1), materials amount and waste (G2), energy consumption (G3), and safety impacts (G4)^34^. The proposed analyzer expressed the highest green score (89.1) compared to the reported methods (48–71.8); Table 3. Ethanol was used for sample preparation, dilution, and extraction. It was recognized by the FDA as a safer alternative to many organic solvents^52,53^ which supported achieving satisfactory G1. Small amounts of reagents and samples, along with reduced volumes of stock solutions and mobile phase, were employed (High G2). The method primarily relied on a smartphone as a highly energy-efficient tool (Energy consumption category 0)^54^(High G3). Additionally, the utilized solvents exhibited low NFPA hazards scores, enhancing users’ safety (High G4). The obtained green score conforms with the conclusion of the previously evaluated moGAPI metric which also highlighted the superior greenness of the developed analyzer.

The blue category evaluates the method’s practicality and applicability, by assessing cost (B1), time efficiency (B2), samples and advanced facilities requirements (B3), and potentiality of miniaturization, automation, and portability (B4)^34,40^. As shown in Table 3, the proposed analyzer obtained the highest blue score of 95.8, compared to the reported methods scores ranging from 28.3 to 73.1. Its construction relied on low-cost materials (˂ $5) (High B1). It required minimal analysis time for the entire assay (High B2). It utilized minimal sample amount, without the need of advanced or sophisticated instruments (High B3). In addition, these evaluated criteria in B2, and B3 together with R1, G2, and G3 significantly contributed to the high score of B1. Moreover, this simple analytical technique was implemented on a portable miniaturized platform (High B4). Consequently, the developed portable at-line analyzer demonstrated an overall whiteness score of 84.8 (Table 3 & Supplementary Fig. S3). In addition, Table 4 showed that the proposed method ranked first among the reported ones. This guarantees the superiority of the proposed method regarding sustainability.

Considering all this, evaluations using moGAPI, CACI, VIGI, and RGB-12 confirmed the superior greenness, practicality, innovation, and overall whiteness of the developed platform.

**Table 3:** Multidimensional sustainability comparative assessment of the proposed and reported methods for TZN determination in its combinations.

	MoGAPI	CACI	VIGI	RGB-12	Ref.
Proposed method					Current work
Reported method 1					^55^
Reported method 2					^56^
Reported method 3					^57^
Reported method 4					^58^
Reported method 5					^59^
Reported method 6					^60^
Reported method 7					^6^

**Table 4:**
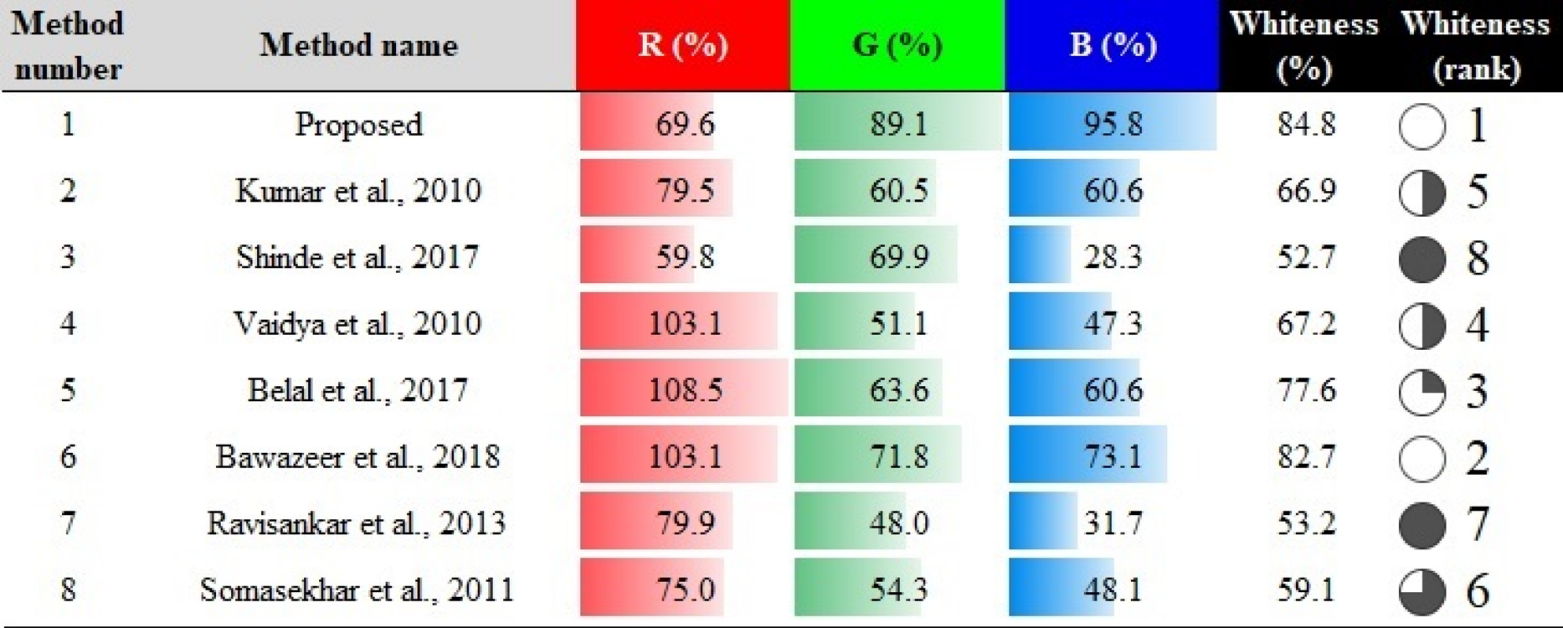
Comparative whiteness evaluation highlighting the ranks of the proposed and reported methods.

Collectively, the proposed work presents a novel approach for cleaning validation. Differentiating from the reported methods **(**Table 5**)**, the developed smartphone-based TLC method combined with either Color Analyzer or ImageJ processing techniques showed distinct capability and reliability to be integrated into a portable at-line analyzer in TZN cleaning validation.

**Table 5:** Comparison of the proposed and reported methods for TZN determination.

Analytical technique	Matrix	Assayed drugs	Correlation coefficient (*r*)	Application	Portable (yes/no)	Ref.
HPTLC / UV detection	Standard solution	Tizanidine Rofecoxib	0.9946 0.9976	Pharmaceutical (tablets)	No	^61^
HPTLC / UV detection	Standard solution	Tizanidine Valdecoxib	0.9996 0.9995	Pharmaceutical (tablets)	No	^62^
HPTLC / UV detection	Standard solution	Tizanidine Nimesulide	0.9983 0.9945	Pharmaceutical (tablets)	No	^63^
HPTLC / UV detection	Standard solution	Tizanidine Aceclofenac	Close to unity (not specified)	Pharmaceutical (tablets)	No	^56^
RP-HPLC / UV detection	Standard solution	Tizanidine Aceclofenac	0.9998 0.9999	Pharmaceutical (tablets)	No	^55^
RP-HPLC / UV detection	Standard solution	Tizanidine Aceclofenac Paracetamol	0.9998 0.9998 0.9999	Pharmaceutical (tablets)	No	^57^
Micellar liquid chromatography / UV detection	Standard solution/plasma/urine	Tizanidine Aceclofenac Paracetamol Nimesulide	0.9999 0.9999 0.9999 0.9999	Pharmaceutical (tablets)/bioanalytical	No	^58^
UPLC / PDA detection	Standard solution	Tizanidine Aceclofenac Paracetamol Nimesulide	0.9999 0.9997 0.9999 0.9999	Pharmaceutical (tablets)	No	^59^
RP-HPLC / UV detection	Standard solution	Tizanidine Aceclofenac Paracetamol Chrozoxazone	0.9996 - - -	Pharmaceutical (tablets)	No	^60^
HPLC / UV detection	Standard solution	Tizanidine Aceclofenac Paracetamol	0.9962 0.9998 0.9948	Pharmaceutical (tablets)	No	^6^
RP-HPLC / UV detection	Standard solution	Tizanidine Ibuprofen	0.9983 0.9996	Pharmaceutical (tablets)	No	^5^
Smartphone-based TLC- color analyzer	Stainless steel surface residues	Tizanidine Aceclofenac Paracetamol Ibuprofen	0.9993 0.9994 0.9963 0.9982	Cleaning validation	Yes	Current work
Smartphone-based TLC-ImageJ	Stainless steel surface residues	Tizanidine Aceclofenac Paracetamol Ibuprofen	0.9990 0.9995 0.9991 0.9992	Cleaning validation	Yes	Current work

## Conclusion

The present work is the first to integrate a portable at-line smartphone-based TLC platform for cleaning validation of TZN in combination with ACF, IBF, and PRC. It provides a simple miniaturized analytical platform that enables rapid detection and accurate quantification of drug residues directly at the equipment site without disrupting manufacturing operations. The proposed platform employed two image processing methods, namely Color Analyzer and ImageJ. They produced satisfactory regression models (*r* > 0.99) over concentration ranges of 0.02–0.17, 1.0–8.5, 4.0–34.0, and 5.0–42.5 mg/mL for TZN, ACF, IBF, and PRC swab samples, respectively. Both methods demonstrated excellent sensitivity. They allowed reliable assay of swab samples, achieving QL approximately 97% − 99% lower than the established acceptance limits. The ImageJ-based approach exhibited slightly improved accuracy and robustness in addition to its ability to generate representative chromatograms. This makes it a reliable substitute for conventional methods when informative and dependable quantitative analysis is required. The Color Analyzer-based approach offered enhanced simplicity and time efficiency, further facilitating effective screening through the generated color density charts. Furthermore, the developed method successfully fulfilled the environmental and sustainability criteria. Overall, the developed portable at-line analyzer represents a highly practical tool for routine cleaning validation of high-risk drugs.

## Supplementary Information

Below is the link to the electronic supplementary material.

Supplementary Material 1

## Data Availability

All data generated or analyzed during this study are included in this published article and in its supplementary information file.
