# Supplementary material for: Sustainable miniaturized smartphone-coupled TLC platform for innovative cleaning validation: application to tizanidine combinations under challenging concentration ratios
Source: Sci Rep. 2026 Jul 19;16:22559. doi: 10.1038/s41598-026-62219-6 (PMC13381885; doi:10.1038/s41598-026-62219-6)
Supplement: Supplementary file 1 — Supplementary Material 1 [file 41598_2026_62219_MOESM1_ESM.pdf]

Supplementary Table S1. Input variables, calculated MACO values, and corresponding AL values for TZN, ACF, IBF, and PRC in combined formulations

	**TZN**	**ACF**	**IBF**	**PRC**
TD (mg)	8.0	400.0	1600.0	2000.0
SF (Oral preparations)			1/1000	
BS (mg)			15,000,000	
LDD (mg)			5000.0	
**MACO (mg)**	24.0	1200.0	4800.0	6000.0
R			0.8	
As (cm^2)			100.0	
At (cm^2)			2519.0	
F			1.0	
V (mL)			0.5	
**AL (mg/mL)**	1.52	76.22	304.88	381.10

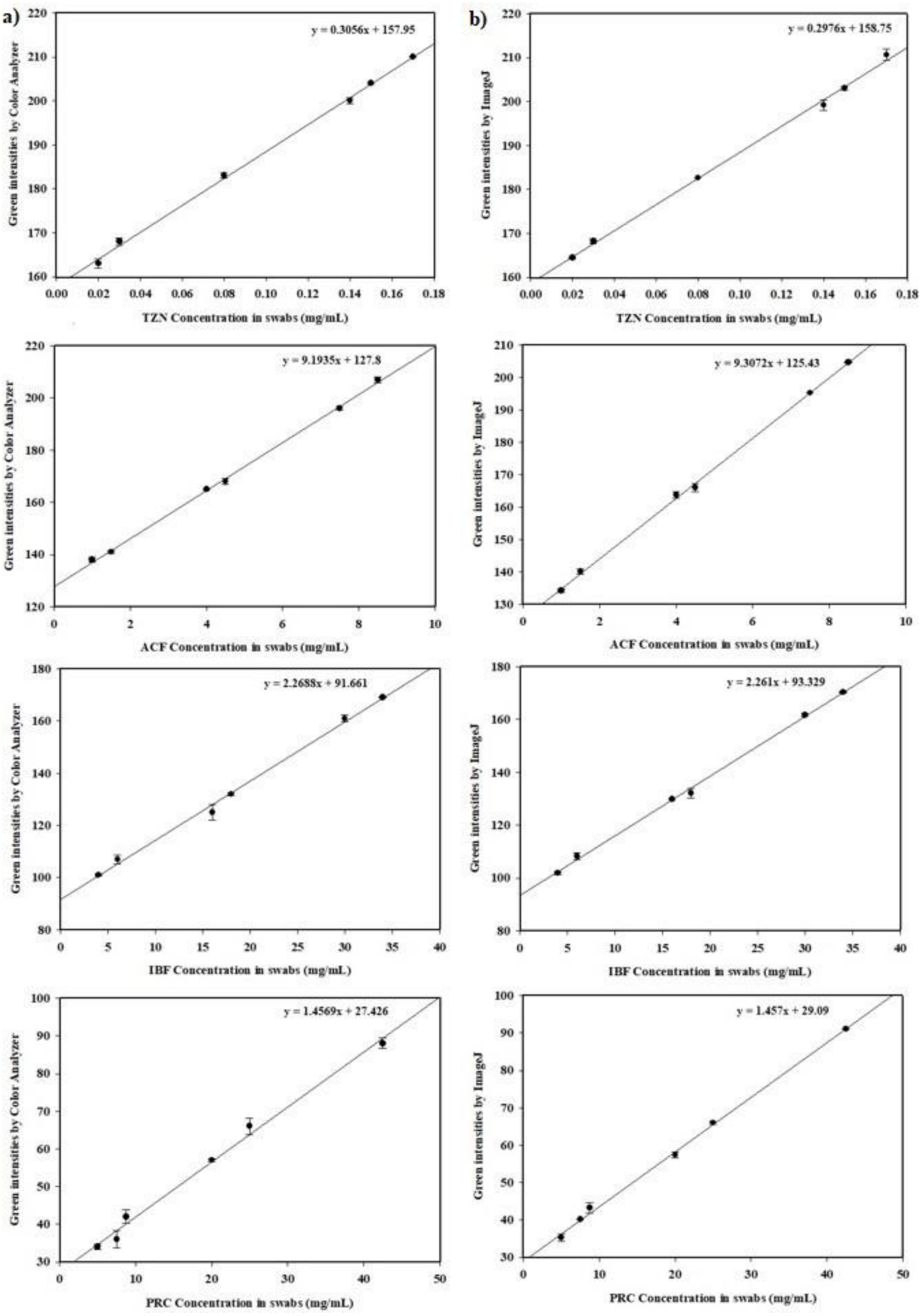

Supplementary Fig. S1. Calibration curves of the proposed method using: **a)** Color Analyzer and **b)** ImageJ

Supplementary Table S2. Precision and accuracy of the proposed smartphone-based TLC combined with Color Analyzer and ImageJ methods for TZN, ACF, IBF, and PRC determination in swab samples

	**Smartphone-based TLC-Color Analyzer**				**Smartphone-based TLC-ImageJ**			
	**TZN**	**ACF**	**IBF**	**PRC**	**TZN**	**ACF**	**IBF**	**PRC**
%RSD [a]	4.22	2.34	1.87	2.04	2.68	1.72	1.35	1.46
%RSD [b]	5.40	3.21	2.66	2.54	4.41	3.0	2.13	3.08
%Recovery [a]	100.83	98.99	100.87	100.48	102.28	98.13	97.42	101.67
%Recovery [b]	103.82	98.65	101.58	98.90	98.25	101.91	97.37	95.67

[a] Intra-day precision (%RSD) and accuracy (%Recovery) were calculated as the average of three concentrations, each analyzed in triplicate (n = 9)

[b] Inter-day precision (%RSD) and accuracy (%Recovery) were calculated as the average of three concentrations, each analyzed in triplicate over three consecutive days (n = 9)

Supplementary Table S3. Robustness of the proposed smartphone-based TLC combined with Color Analyzer and ImageJ methods for TZN, ACF, IBF, and PRC determination in swab samples

	**Smartphone-based TLC-Color Analyzer**				**Smartphone-based TLC-ImageJ**			
	**TZN**	**ACF**	**IBF**	**PRC**	**TZN**	**ACF**	**IBF**	**PRC**
**Condition**	**%RSD [a]**							
Swab size	4.86	0.51	1.83	4.89	0.97	4.39	4.03	1.42
Extraction solvent volume	5.38	4.19	3.71	1.12	1.23	0.99	5.56	4.60
Mobile phase ratio	4.52	1.82	4.58	2.53	5.13	3.41	2.58	2.59
Imaging distance	4.39	0.87	1.33	2.06	3.70	0.65	3.57	5.52

[a] %RSD results were expressed as the mean of three determinations for each condition (n = 9)

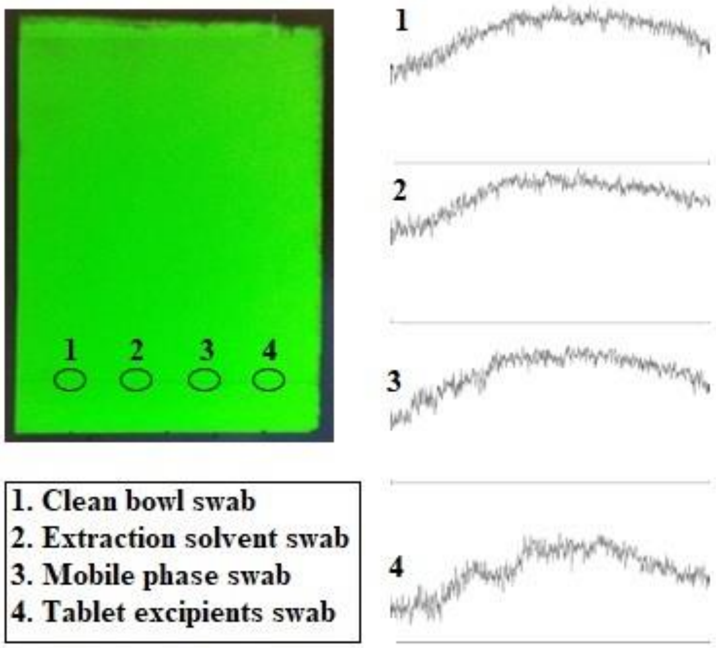

Supplementary Fig. S2. Developed TLC plate and the corresponding ImageJ generated chromatogram of swabs of clean bowl and others containing potentially interfering substances

Supplementary Table S4. Recovery study of the proposed smartphone-based TLC combined with Color Analyzer and ImageJ methods for TZN, ACF, IBF, and PRC in swab samples

	**Smartphone-based TLC-Color Analyzer**				**Smartphone-based TLC-ImageJ**			
	**TZN**	**ACF**	**IBF**	**PRC**	**TZN**	**ACF**	**IBF**	**PRC**
**Concentration level**	**%Recovery ± %RSD [a]**							
Low [b]	91.09 ±6.66	94.17 ±4.26	92.61 ±2.57	90.29 ±9.02	93.00 ±8.84	94.17 ±2.19	94.44 ±0.77	91.03 ±3.54
Mid [c]	94.13 ±2.72	92.20 ±1.01	95.59 ±1.79	90.52 ±5.53	94.45 ±0.18	92.75 ±1.45	95.02 ±0.76	91.54 ±3.25
High [d]	96.68 ±0.94	95.03 ±2.03	96.73 ±1.60	90.30 ±1.32	95.78 ±1.48	96.83 ±0.42	97.11 ±2.09	92.70 ±1.61

[a] %Recovery and %RSD were expressed as the mean of three determinations of each concentration level (n = 3)

[b] Low concentration level was 0.02, 1.0, 4.0, and 5.0 mg/mL for TZN, ACF, IBF and PRC, respectively.

[c] Mid concentration level was 0.10, 5.0, 20.0, and 25.0 mg/mL for TZN, ACF, IBF and PRC, respectively.

[d] High concentration level was 0.17, 8.5, 34.0, and 42.5 mg/mL for TZN, ACF, IBF and PRC, respectively.

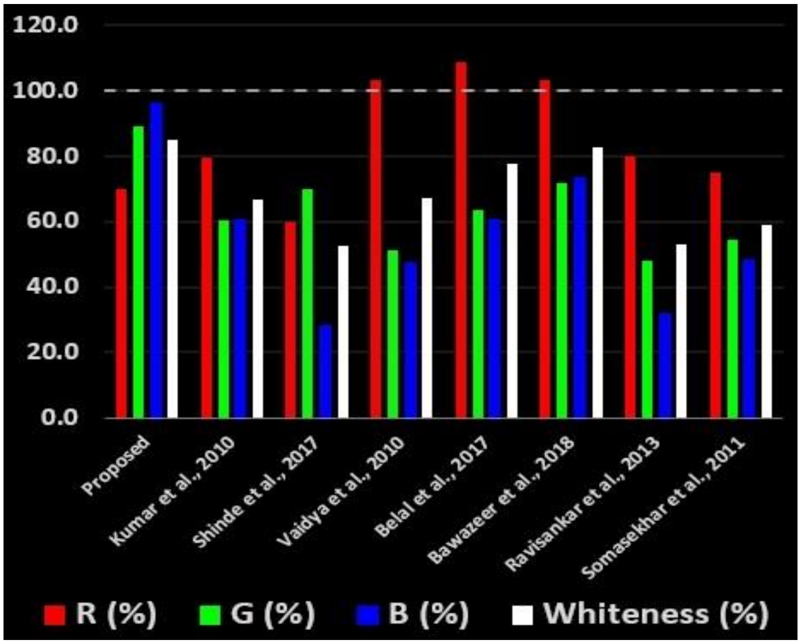

Supplementary Fig. S3. Whiteness relative ranking bar chart of the proposed and reported methods
